# Monitoring metal patterns from urban and agrarian sites using the bumblebee *Bombus terrestris* as a bioindicator

**DOI:** 10.1007/s11356-023-30504-w

**Published:** 2023-11-07

**Authors:** Leonie Rabea Breidenbach, Lena Benner, Martina Roß-Nickoll, Volker Linnemann, Andreas Schäffer

**Affiliations:** 1 Institute for Environmental Research, RWTH Aachen University, Worringerweg 1, 52074 Aachen, Germany; 2 Institute of Urban Water Management, RWTH Aachen University, Mies-Van-Der-Rohe-Str. 1, 52074 Aachen, Germany

**Keywords:** Biomonitoring, Bioaccumulation, Boron, Cadmium, Bumblebee

## Abstract

Honeybees are well-established bioindicators for different types of pollutants. This study aims to establish another species of the Apidae family as a bioindicator, with a distinct behaviour and life cycle. The bumblebee *Bombus terrestris* was used as a bioindicator for 12 metals. Bumblebee hives were placed at sampling sites in and around the city of Aachen, Germany, and metal concentrations were assessed using ICP-MS. Metal concentrations were compared to those found in honeybees described in the literature. Spatial differences in metal patterns were investigated by comparing two land-use types: urban and agrarian. Seasonal differences were compared by taking samples in spring and summer. All analysed metals were detected above the detection limit and within or even above the concentration range found in honeybees. Significant spatial differences were found for the metalloid B and the metal Cd with higher concentrations at the agrarian sites than the urban sites. Significant seasonal differences were found for 8 metals: Fe, Zn, Cu, Ni, Cd, and As concentrations were higher in summer than in spring, while B and V concentrations were higher in spring. To categorise the results, we applied the honeybee contamination index (HCI) and adapted it to bumblebee purposes. According to the HCI, only one agrarian site showed a high contamination level. This study shows that bumblebees are suitable bioindicators for metals. The obtained data can serve as a first baseline in the establishment of additional monitoring studies or risk assessments.

**Supplementary Information:**

The online version contains supplementary material available at 10.1007/s11356-023-30504-w.

## Introduction

Not only since the publication of Hallmann et al. (2017), detecting a 75% decline of the biomass of all flying insects in Germany, the importance of studying the underlying mechanisms of the insect decline became apparent. Insects are not only crucial to maintain ecosystem functions such as the preservation of the gene flow among populations of flowering plants, but they also provide essential ecosystem services such as pollination, pest control or the nutrient cycle as they decompose different kinds of biological materials (Losey and Vaughan 2006). Three quarters of all crop plants are pollinated by insects (Vanbergen 2013).

Although the headword “insect decline” results in 1,220,00 scientific articles (Google Scholar), the origin of the insect decline remains uncertain. There are indications that the decline already began in the 1950s, simultaneously to the beginning of the anthropocene (Wagner 2020) pointing to the assumption that it is linked up to man-made activities. As human beings altered almost every natural habitat for cultivation or construction purposes, insects can hardly find sufficient refugium (Potts et al. 2010). Other possible reasons, additional to the land use change with the consequence of habitat loss and fragmentation, are the following: introduced alien species, the spread of pathogens (Genersch et al. 2010), climate change (review by Harvey et al. 2022), decreased resource diversity and anthropogenic pollutants such as agrochemicals (Potts et al. 2010, review by Wagner 2020). However rather than only one of these factors, it is likely to be a combination of stressors (Potts et al. 2010). To combat these, it is crucial to identify every single threat, to apply a comprehensive approach to prevent insects from extinction.

One possible driver of the insect decline is anthropogenic pollutants reaching the environment (Wagner 2020). Insects are exposed to anthropogenic pollutants as they are emitted ubiquitously around the world and distributed even to the most remote places like Antarctica. Xenobiotics like polycyclic aromatic hydrocarbons (PAHs), polychlorinated biphenyls (PCBs), perfluorinated compounds (PFCs) or metals are emitted and introduced into different ecosystems, accumulate there and/or cause adverse effects to biota of all trophic levels (Mateo et al. 2016; Papa et al. 2021; Ilijević et al. 2021). Panagos et al. (2013) referred to heavy metals as “the main pollutants in European soils and groundwater” (Panagos et al. 2013; Vareda et al. 2019). Not only mammals are threatened by metal exposure, but adverse effects have also been shown in insects. That is one of the reasons to further investigate this specific group of pollutants within this study, which is done by using a living organism as a bioindicator.

The buff-tailed bumblebee *Bombus terrestris* is a native pollinator in Europe that lives in annual, underground colonies with up to 500 individuals. In early spring, the gynes found new colonies and carry out all necessary tasks until enough new workers have hatched to take over. Therefore, the queen regularly leaves the hive where she can come into contact with anthropogenic pollutants such as pesticides or metals. During their foraging flights, workers fly distances of up to 800 m but prefer to stay close to the hive (100 m) if enough food sources are available (Walther-Hellwig and Frankl 2000; Wolf and Moritz 2008). They forage at temperatures of above 7 °C, enabling them to forage earlier in the year as honeybees and in colder regions, such as the tundra. Bumblebee colonies are commercially available for use in greenhouses or fruit orchards. Each forager performs > 10 foraging flights per hour (Arce et al. 2017), visiting hundreds to thousand flowers per day. During the last years, several guidelines and test designs have been published to assess the effects of pollutants on *B*. *terrestris*. Thus, it is a good novel model organism for pollinators, representing a compromise between handling as a test organism (sufficient number of individuals, commercially available, active period) and similarity to wild bee species (annual life cycle, small numbers).

To our knowledge, this is the first study to use the bumblebee *Bombus terrestris* as a bioindicator for metals. Due to its unique morphology and life cycle, it can “collect” pollutants and at the same time display the amount of pollutants insects are exposed to during their life. This study attempts to evaluate the suitability of *B*. *terrestris* as a bioindicator for metal contamination. Different anthropogenic activities at different land-use types, such as fertiliser and sewage sludge application at agrarian sites and fuel combustion or tyre and break abrasion at urban sites, might lead to characteristic metal patterns to be found in bumblebees. To evaluate and compare these specific patterns, six sites at urban and agrarian land-use types were selected for the bumblebee hives. Ten metals, the metalloid arsenic and the semimetal boron, were chosen for the comparison of concentration differences in the bumblebees. For the sake of simplicity, all 12 substances will be referred to as “metals” in the following text. As it was shown in various studies that there is a seasonal influence on metal concentrations to be found in bioindicators, likely to result from different weather phenomena, this study also aims at comparing two different sampling seasons (spring and summer) to evaluate whether the season has an influence on the metal content in bumblebees.

## Material and methods

*Bombus terrestris* hives (each with queen, ~ 50 workers and brood) were bought from the breeder company Koppert (German supplier: Landhandel Pegels, 47918 Tönisvorst; Dutch supplier: Mertens B.V., 5960 AC Horst) which usually produces bumblebee colonies for pollinating crops, cultivated in greenhouses. One hive from each supplier was directly frozen, without contact to the outside world and its pollutants. Bumblebees from these hives were used as control, to determine metal background concentrations.

### Sampling sites

**Fig. 1:**
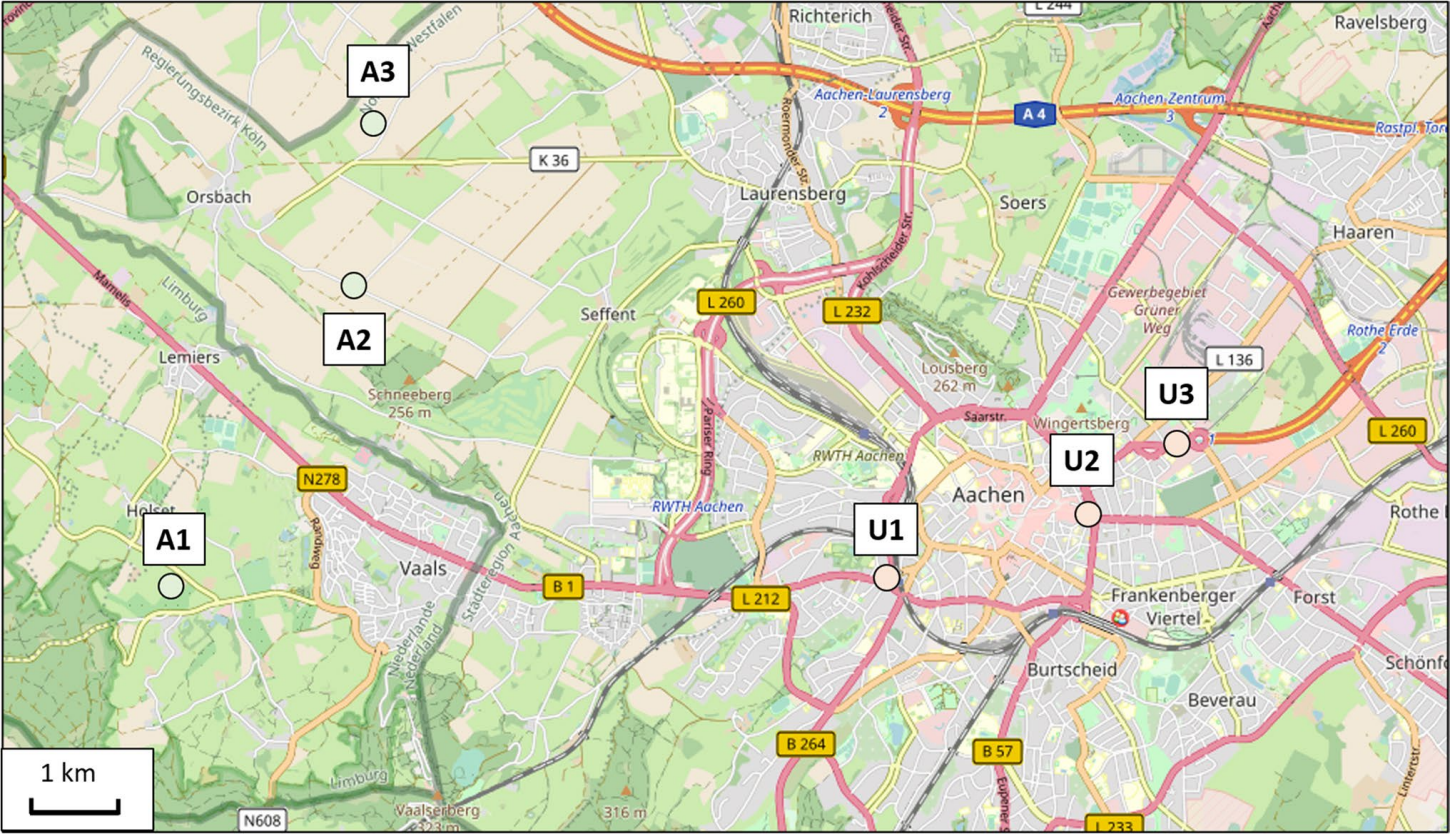
An overview map of the six bumblebee hive locations. The three agrarian sites, indicated by (A) on the west side and the three urban sites, indicated by the letter (U), located within the city of Aachen on the east side. The green line indicates the German–Dutch border. Source of map: open street map (FOSSGIS e.V. 2001)

Three representative agrarian sites close to Aachen (western Germany, on the border to the Netherlands) were chosen by virtue of their proximity to cultivated crop areas. Three urban sites within the city of Aachen were chosen due to their proximity to busy roads with high traffic volumes. A map with an overview of the bumblebee hive locations is shown in Fig. 1.

The location A1, which is located on the ground of a fruit orchard (apples, cherries, strawberries), is located in the Netherlands, close to the German border (50.770640, 5.990961). At location A2, the bumblebee colony was placed between a field and a narrow asphalt road on an overgrown small slope (50.791367, 6.011156). The road next to the hive is mainly used by cyclists, rarely by cars. A3 was located between a cornfield and a meadow (50.801599, 6.010808) with no big road nearby. The urban sites were all located in the centre of Aachen city. U1 was placed next to the railway station “Schanz” (50.770472, 6.073227) where local trains pass by frequently. It was located on a small green space, next to a big crossroad with highly frequented traffic. U2 was located on the crossway “Kaiserplatz” (50.775281, 6.095542), next to one of Aachen’s main arteries, a double-tracked federal highway, where thousands of commuters pass by every day. U3 was located at the parking area “Blücherplatz” (50.779621, 6.103631), close to a double-tracked federal highway as well as a petrol station.

### Sampling dates and procedure

At each of the six sites, three bumblebee hives were placed from April to July, and two new hives were placed from July to September. The hives located next to each other were considered one unit so that bumblebees were taken from either one of them. The hives were placed around half a metre above the ground in waterproof housings to avoid fouling. Inside the hives, a sugar solution ensured the food base of the bumblebees. Samples were taken in April (first set of hives) and July (only from newly installed hives), to avoid effects due to pollutant accumulation in the hive itself. In total, 30 bumblebee samples were analysed, consisting of three control samples (two from a breeder in the Netherlands and one from a breeder in Germany), 14 samples from agrarian sites and 13 samples from urban sites. Every of the three agrarian sites (A1, A2, A3) as well as the three urban sites (U1, U2, U3) was represented with 4 to 5 samples each, divided into two to three samples per sampling date (see table S1).

Bumblebees returning from their forage flights were captured in front of the hives. This procedure was used to ensure that the bumblebees left the hive at least once to possibly be exposed to metals during their flights. Bumblebees were captured using a brailer which was cleaned before with methanol. After capturing the bumblebees, they were transferred into a glass vial and put into liquid nitrogen to freeze the bumblebees. Until returning to the laboratory, the bumblebee samples were stored in a cooling box containing cold packs.

In the laboratory, they were stored in an − 26 °C freezer until further treatment. Every bumblebee was weighted, and the pollen on their legs was removed and weighted as well. For the metal extraction, only the bumblebees were extracted.

### Metal extraction

In the absence of a suitable extraction method for bumblebees, the metal extraction followed the procedure established by Goretti et al. (2020) for honeybees. Therefore, 1 g fresh weight of bumblebee was used. In most cases between one and seven individuals, bumblebees were pooled as one sample. The bumblebees were dried for 16 h at 105 °C in a drying oven. The dry weight of every bumblebee was measured afterwards. The mean weight loss after drying was 69.2% so that ca. 310 mg of dry bumblebee was finally extracted.

The vessels and equipment used for the extraction were cleaned with nitric acid (10%) for a 24 h period and afterwards rinsed with Milli Q water several times to avoid metal contamination. For the acid digestion, the bumblebee samples were put into the microwave vessels, and 8 mL of nitric acid (65% v/v) and 2 mL of hydrogen peroxide (30% v/v) were added. The extraction occurred at 170 °C for 30 min in a microwave unit (µPREP-A, MWS star T-SYSTEME, MLS GmbH, Leutkirch im Allgäu, Germany). Subsequently, the remaining suspension was cooled down at room temperature for ca. 1.5 h. The result of the microwave extraction was a clear solution with no visible particles. This solution was then filtered (2.5 µm filter, Rotilabo-Rundfilter qualitative, Roth Karlsruhe) and diluted to 25 mL with Milli Q water. For the bumblebees of the sample date in April, nitric acid for analysis (Emsure, Reag. Ph Eur, ISO) was used for the extraction. For the July samples, suprapure nitric acid was used (Merck, Germany).

### Metal analysis

The following twelve metals (and metalloids) were investigated: Aluminium (Al), Arsenic (As), Lead (Pb), Boron (B), Cadmium (Cd), Chrome (Cr), Iron (Fe), Copper (Cu), Nickel (Ni), Vanadium (V), Zinc (Zn), and Mercury (Hg). Boron and arsenic are metalloids and not metals, but for simplification, hereafter, the term “metals” will include all twelve analysed metals. These twelve specific metals were chosen either because of their high toxicity, their high concentrations to be found in environmental compartments or interesting findings in preliminary tests.

Metal analysis was carried out using an inductively coupled plasma mass spectrometer (ICP-MS, Agilent Technologies, Ratingen, Germany, Agilent SYS-IM-7700) using argon gas for the plasma generation. Inside the plasma a temperature of 10.000 K was generated. The detection limits for the investigated metals varied from 0.01 µg/l for Hg to 0.1 µg/l for Cd, Cr, Cu, Ni, Pb, As, and V to 1 µg/l for Zn, Fe, Al, and B. Three internal added standards were used for calibration: Scandium (5 mg/L), Indium (0.2 mg/L), and Lutetium (0.2 mg/L).

The blank solution contained 1% of HNO_3_ and 0.5% of HCl in Milli Q water. For result evaluation, the Software “MassHunter” version B.01.02 from Agilent technologies was used.

The metal concentrations obtained in µg/L were transformed into mg/kg dry weight of bumblebee. To analyse normal distribution of control, urban and agrarian sites, Shapiro–Wilk Normality test was applied using the program RStudio Version 1.4.1103 (2009–2021, RStudio, PBC, Joseph J. Allaire). For testing on homogeneity of variances, the Bartlett test was applied. As almost all of the data were either not normal distributed or not homogenic in variance, the Mann–Whitney U test was used to analyse the significance of spatial differences between control, agrarian and urban sites. The same test was used for investigating the significance of temporal differences between sampling dates April and July at the level of *p* < 0.05, *p* < 0.02 and *p* < 0.005.

### Honeybee Contamination Index

To generalise the results and get a better understanding of the contamination level of the bumblebees, the Honeybee Contamination Index (HCI), developed by Goretti et al. (2020), was applied and transformed for bumblebee purposes. The HCI allows the results to be classified into three classes: high, intermediate and low contamination level. Therefore, two reference threshold values were used to represent low and high contamination. The threshold values were derived from Gutiérrez et al. (2015), who calculated the reference values based on experimental data of honeybees, classifying results into quartiles which define low and high reference values. As those threshold values are given as mg/kg wet weight, it was converted into dry weight bumblebee assuming an average 69.2% weight loss after drying (data calculated from the bumblebee weight difference before and after drying in this study). Original and converted reference values are shown in Table 1.

**Table 1:** Reference values for honeybee contamination index obtained from Gutiérrez et al. (2015) in mg/kg wet weight (ww) and converted reference values into mg/kg dry weight (dw) bumblebee, assuming a 69.2% weight loss

	Low reference value		High reference value	
Metal	(mg/kg ww)	(mg/kg dw)	(mg/kg ww)	(mg/kg dw)
Cd	0.05	0.16	0.1	0.32
Pb	0.3	0.97	0.7	2.27
Cr	0.04	0.13	0.12	0.39
Ni	0.1	0.32	0.3	0.97

The HCI was then calculated by the following equation:

$${HCI}_{\mathrm{1,2}}=log(\frac{{c}_{\mathrm{measured}}}{{c}_{\mathrm{reference1,2}}})$$

with *c*_measured_ being the concentration measured in the bumblebee and *c*_reference_ either being the high reference value (*c*_reference1_) or the low reference value (*c*_reference2_). A positive HCI_1_ indicates a high contamination level, a negative HCI_2_ a low contamination level and a negative HCI_1_ and a positive HCI_2_ indicates an intermediate contamination level.

## Results

### Spatial differences

**Fig. 2:**
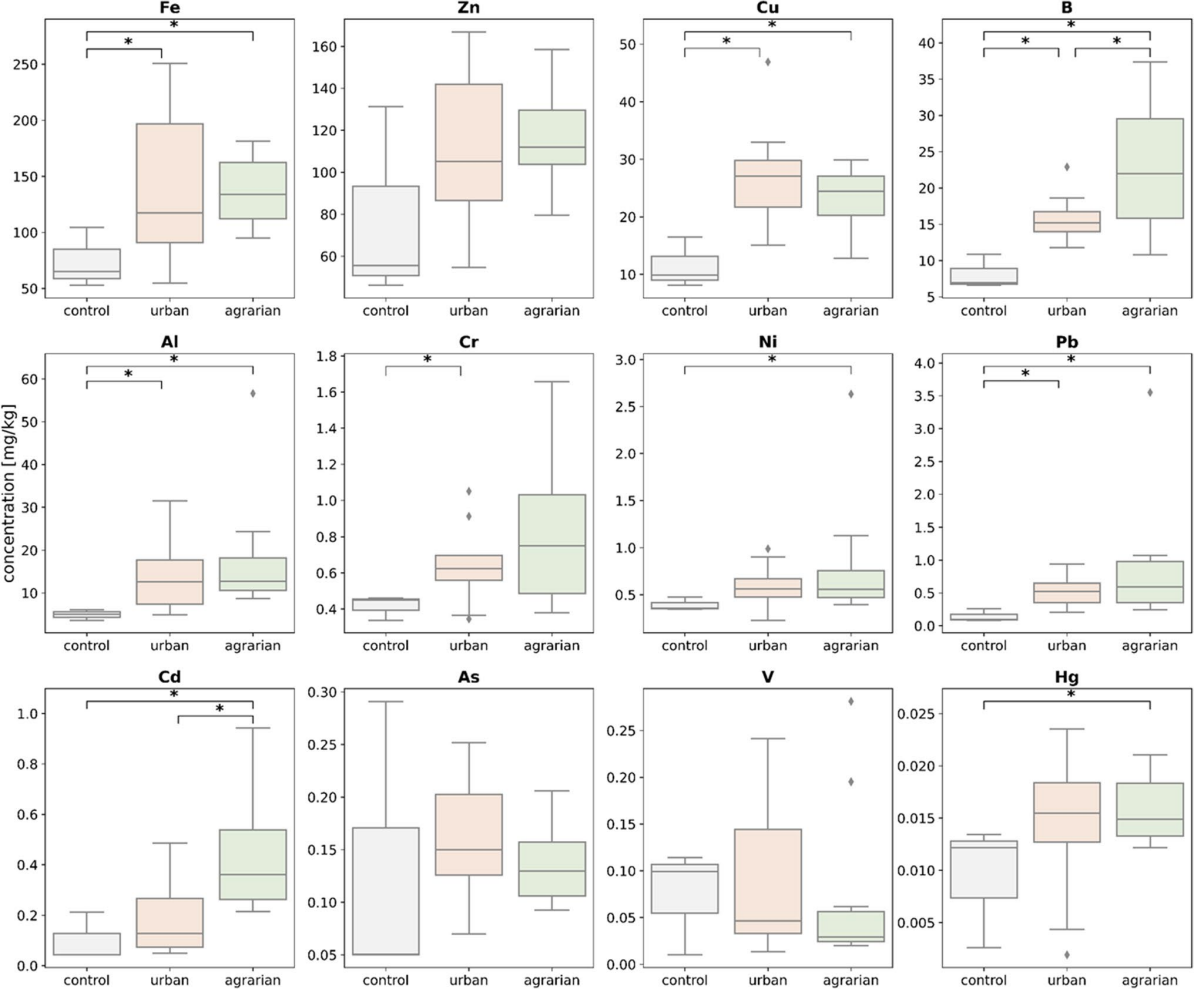
Distribution of the twelve measured metal concentrations in *B. terrestris* (mg/kg dw bumblebee) for the control samples (*n* = 3, white), the samples taken at urban (*n* = 13, orange) and the samples taken at agrarian sites (*n* = 14, green). Significant differences are indicated as follows: *p < 0.05, **p < 0.02, ***p < 0.005

The distribution of metal concentrations for control samples, urban and all agrarian sites (including both sampling dates: April and July) are shown in Fig. 2. The metal with the highest mean concentration found in bumblebees from agrarian sites was Fe (137 mg/kg dw) followed in the declining order by Zn > Cu > B > Al > Cr > Pb > Ni > Cd > As > V, and the lowest concentration was found for Hg (0.02 mg/kg dw). Mean metal concentrations found in bumblebees from urban sites followed the same order except that Pb and Ni were interchanged.

All twelve measured metals showed higher concentrations in samples from field sites compared to the control samples. Significant differences (*p* < 0.05) between the agrarian sites, and the control was found for eight out of the twelve metals (Fe, Cu, Pb, Ni, Cd, Al, Hg and B). Significant differences between the control and the urban sites were found for four out of the twelve metals (Cu, Pb, Al and B).

Significant differences between urban and agrarian sites were found for two metals: Cd and B. For both, significantly higher concentrations were found in the agrarian samples. The mean metal concentrations found in agrarian sites were higher than the urban means for eight out of twelve metals: Zn, B, Al, Cr, Ni, Pb, Cd, Hg. For Fe, Cu, As and V, the urban mean was higher than the agrarian mean.

### Temporal differences

**Fig. 3:**
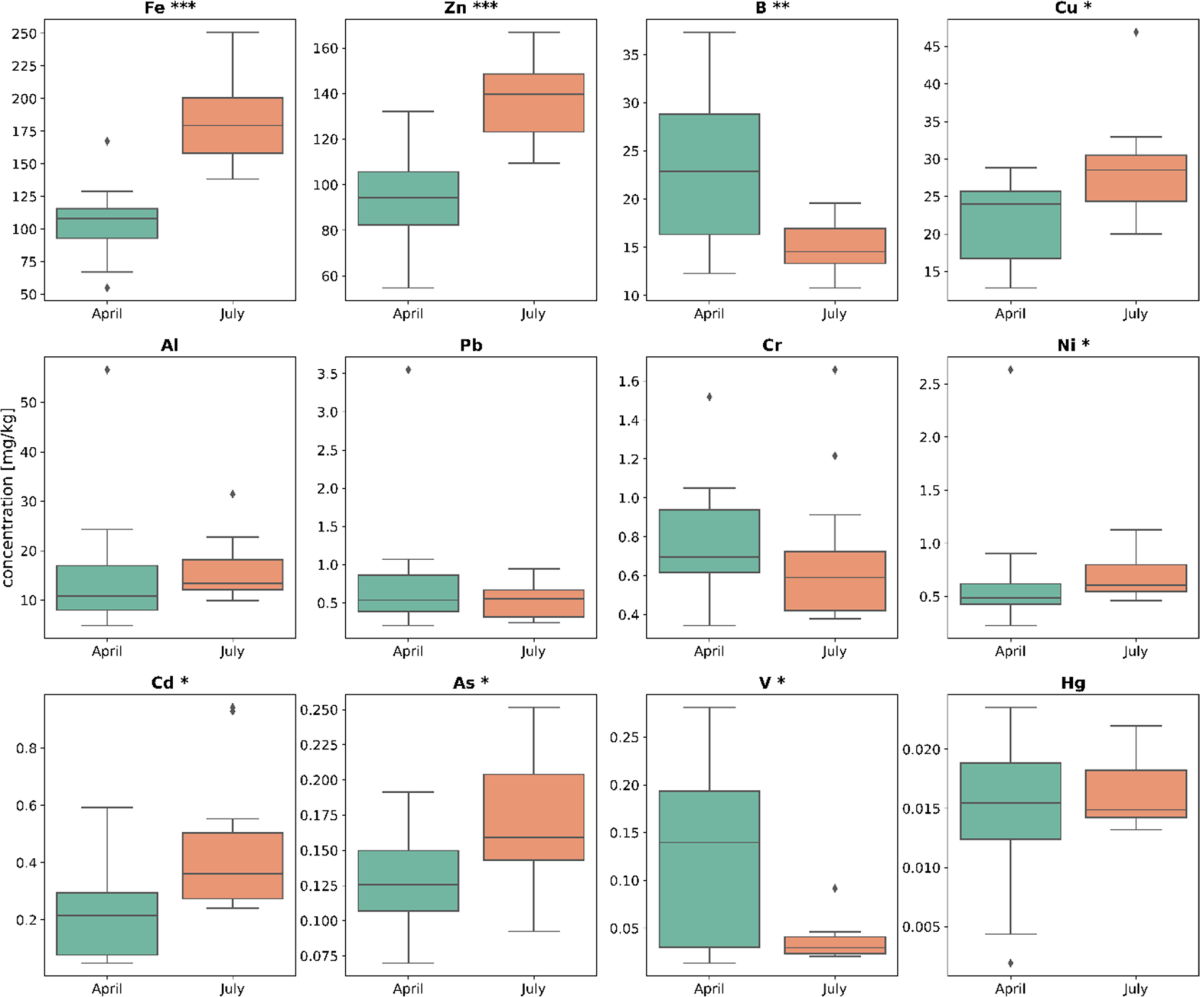
Distribution (shown as boxplots) of the twelve measured metal concentrations in *B. terrestris* (mg/kg dw bumblebee) for the April samples (*n* = 15, green) and the July samples (*n* = 12, orange). Significant differences are indicated as follows: *p < 0.05, **p < 0.02, ***p < 0.005

The overall metal concentrations were higher in July than in April. The distribution of the metal concentrations found in bumblebee samples obtained in April compared to samples obtained in July is shown in Fig. 3. Significant differences in concentrations between the two sampling dates were found for eight out of twelve metals: Fe, Zn, B, Cu, Ni, Cd, As and V. Al, Pb, Cr and Hg did not show significant differences between April and July samples.

**Fig. 4:**
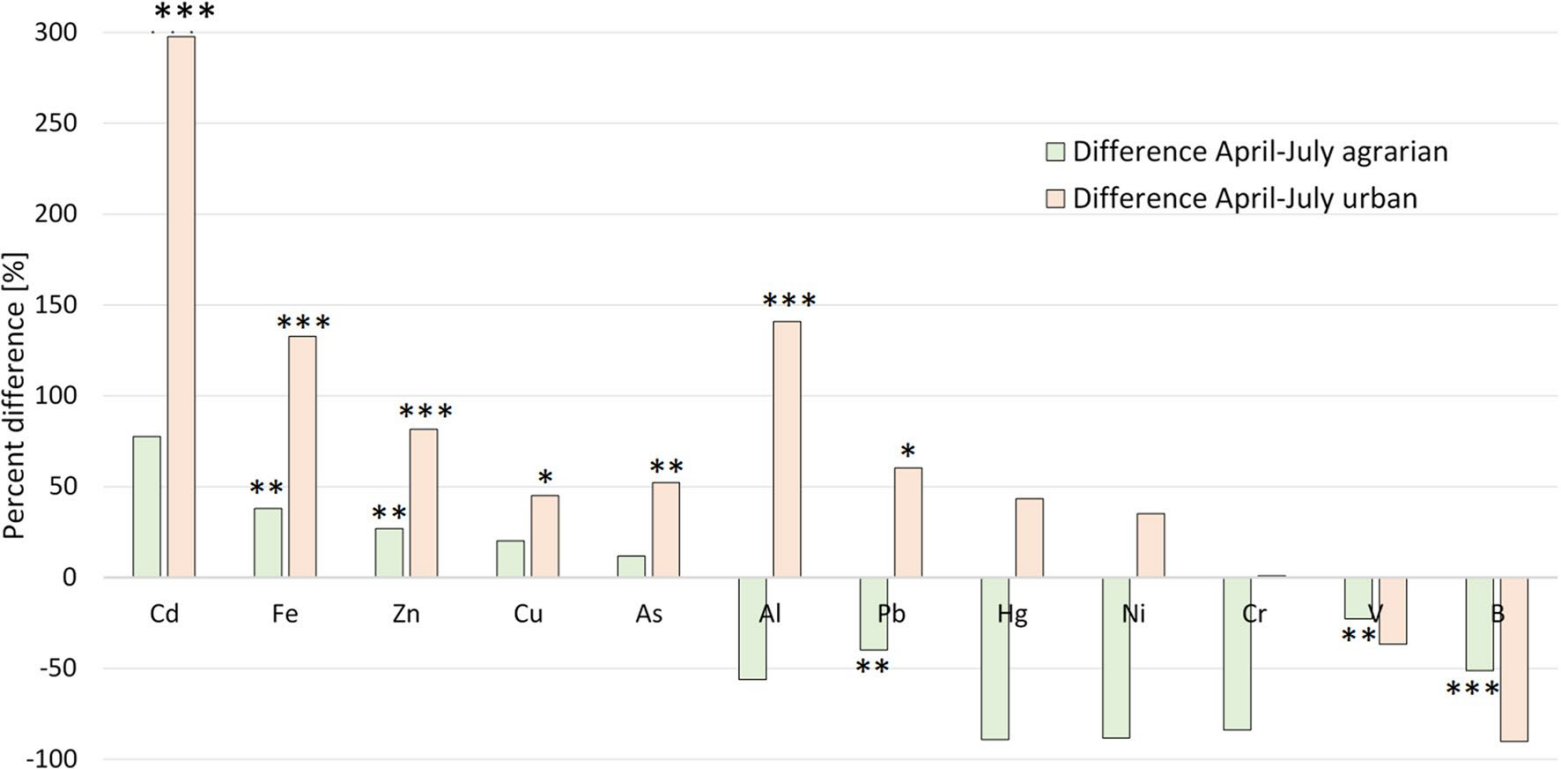
Difference in percent of metal concentration in *B. terrestris* between April and July. Percent differences are shown for the agrarian sites (green) and the urban sites (orange). Positive values indicate an increase and negative values a decrease in concentration. Significant differences are indicated as follows: *p < 0.05, **p < 0.02, ***p < 0.005

Relative differences in metal concentration between April and July for urban and agrarian sites are shown in Fig. 4. Significantly higher concentrations for July compared to April at agrarian sites were found for Fe (38%) and Zn (27%). For April, significantly higher concentrations were found for Pb (40%), V (23%) and B (51%). At urban sites, significantly higher metal concentrations in July compared to April were found for Cd (298%), Fe (133%), Zn (82%), Cu (45%), As (52%), Al (141%) and Pb (60%). No metal showed significantly higher concentrations for April than in July at urban sites.

### Honeybee Contamination Index (HCI)

**Fig. 5:**
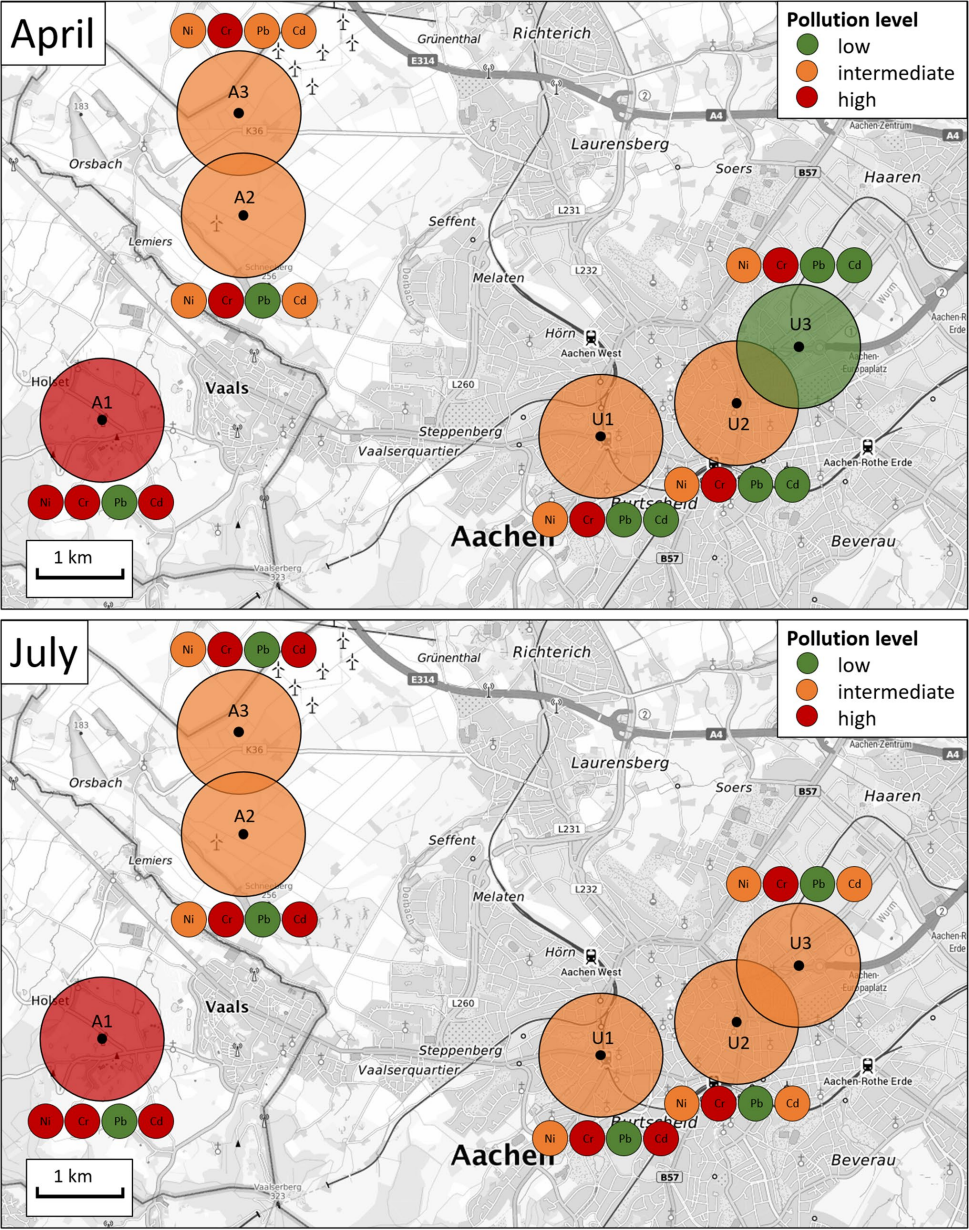
Pollution levels of the six analysed sites (agrarian (A) and urban (U)) in April (top) and July (bottom) calculated with the Honeybee Contamination Index (HCI). The big circles represent the mean forage areas of bumblebees, and the colour indicates the mean pollution level at this site. The small circles indicate the pollution level for each site for either Nickel (Ni), Chrome (Cr), Lead (Pb) or Cadmium (Cd) individually. Green indicates a low, orange an intermediate and red a high pollution level

An overview of the different pollution levels for each specific site for either April or July is shown in Fig. 5. The big circles represent the area one bumblebee covers during a forage flight with a mean radius of ca. 500 m (Osborne et al. 1999). The mean pollution level at each site was calculated as the mean value of the HCIs of the four metals to express a synthetic judgement. In April, two agrarian sites (A2 and A3) and two urban sites (U1 and U2) showed an intermediate pollution level. A1, the site located on the ground of an apple orchard, showed a high level of pollution, whereas the site U3 located at the Blücherplatz within the city of Aachen showed a low mean level of pollution.

In July, mean levels of pollution remained constant at all locations except from U3 which was low in April and changed to intermediate in July. When looking at each of the four metals which contribute to the mean pollution value, separately, it stands out that Cr showed a high pollution level at each of the six investigated sites in April as well as in July. Ni pollution, mainly intermediate except from A1, stayed constant over the sampling period, whereas Cd pollution levels rose from April to July one order of magnitude on every site (except from A1) and at U1 even two orders of magnitude. Pb pollution was low on every site except from A3 where it was intermediate in April; in July, Pb pollution level was low on all six sites. As the HCI is dependent on the number of data points and the resulting threshold values by Goretti et al. (2020), we included the raw data in the SI (table S2 and S3) to enable future studies to develop the HCI based on the specific requirements of bumblebees.

## Discussion

### Heavy metal levels in bumblebees—comparison to concentrations in honeybees

The first objective of this study was to evaluate the suitability of bumblebees as bioindicators for metals, in particular in comparison to the established honeybees. Honeybees are widely used as bioindicators for different substances such as ultrafine particulate matter (Papa et al. 2021), pesticides (Traynor et al. 2021; Benner et al. 2023) and other elements (Zarić et al. 2022a; Ilijević et al. 2021). As there are also various studies available about honeybees as bioindicators for metals, metal concentrations measured in this study were compared to metal data for honeybees from different European countries in the following paragraphs.

Fe and Zn are the metals with the highest concentrations in all honeybee studies which analysed different metals (Fakhimzadeh and Lodenius 2000; Van der Steen et al. 2012; Zarić et al. 2017; Goretti et al. 2020). These two metals are essential micronutrients, and many organisms have regulating mechanisms such as metallothioneins for maintaining a constant concentration within the body (Kägi and Schäffer 1988; Szentgyorgyi et al. 2011). Fe concentrations found in honeybees from other authors range from 63 to 317 mg/kg dw honeybee, whereas it ranged from 53 to 251 mg/kg dw bumblebee in our study. It was shown that honeybees have deposits of Fe within their abdomen which is assumed to serve for orientation among the earth’s magnetic field (Gould et al. 1978; Loper 1985). This could be one explanation for the slightly higher concentrations found for Fe in honeybees as such an Fe deposit is not yet known for bumblebees.

Lindqvist (1993) found similar Fe concentrations (mean concentrations of 120 mg/kg up to 245 mg/kg) in *B. terrestris* queens at uncontaminated sites in Sweden, as in this study. This leads to the assumption that bumblebees have some control mechanisms for the uptake of Fe into their bodies. The significantly lower Fe concentrations in the control bumblebees could indicate an Fe deficit due to the absence in their feeding solution during breeding. All this may suggest Fe to be an essential element for *B*. *terrestris* ingested from certain environmental compartments like nectar. However, further investigation will be necessary like analysing the feeding solution from the breeder on its metal content or monitoring Fe uptake from infused feeding solutions.

Zn concentrations in *B*. *terrestris* queens from uncontaminated sites were measured in median concentrations of 105 mg/kg (Lindqvist 1993), with a large standard deviation of up to 25 mg/kg. As these maximum concentrations exceed the findings in our study (max. 167 mg/kg dw), this leads to the same assumption as for Fe: anthropogenic activity is not the major influence on Zn concentrations in bumblebees. Zn is an essential metal in numerous metalloenzymes and other proteins like metallothionein. However, high Zn concentrations in the environment were found to cause sublethal effects in insects such as decreased body size in black garden ants (Grześ et al. 2015) thus, environmental monitoring should include Zn concentrations.

Al concentrations found in studies using honeybees as bioindicators vary between 4.6 mg/kg in a rural area in the Netherlands (Van der Steen et al. 2012) and 104 mg/kg at an urban site in Serbia (Zarić et al. 2017). The Al concentrations found in this study ranged between 4 mg/kg and 57 mg/kg dw, and thus in a comparable range as the honeybee studies but lower than the exceptionally high value found in Serbia.

Cr concentrations in honeybees were found to be 0.05 mg/kg at uncontaminated sites in Italy (Conti and Botrè 2001) and 3.5 mg/kg at an urban site in Poland (Skorbiłowicz et al. 2018). Most of the studies using honeybees as bioindicators found maximum Cr concentrations below 0.62 mg/kg even at highly urbanised or industrial regions (Conti and Botrè 2001; Roman 2005; Van der Steen et al. 2012; Zarić et al. 2017; Goretti et al. 2020). In this context, Cr concentrations found in this study of between 0.3 and 1.7 mg/kg dw are comparatively high.

Pb concentrations in honeybees vary between 0.003 and 6.73 mg/kg both found at an urban region in Poland (Roman 2010) and between 0.324 and 4.0 mg/kg dw in an urban area in Serbia (Zarić et al. 2018). The low Pb concentration was found in summer and the high concentration in spring. Pb concentrations found in bumblebees from this study varied between 0.08 and 3.6 mg/kg. We also found higher Pb concentrations at agrarian sites in spring, whereas at urban sites, the contrary was found, i.e. higher Pb concentrations in summer.

Concentrations for As found in honeybees vary between 0.04 and 1.64 mg/kg (Van der Steen et al. 2016; Zarić et al. 2022a) which is almost one order of magnitude higher than found in bumblebees of this study (max. 0.29 mg/kg).

 V concentrations in honeybees vary from 0.006 (Van der Steen et al. 2012) to 0.32 mg/kg (Van der Steen et al. 2016), a similar range found in this study of 0.01 to 0.28 mg/kg.

Cd concentrations in honeybees from different land-use types vary between 0.03 (Fakhimzadeh and Lodenius 2000; Goretti et al. 2020) and 4.2 mg/kg (Conti and Botrè 2001), whereas concentrations found in this study varied between 0.04 and 0.9 mg/kg. Cd is actively absorbed by plant roots and transferred via the vascular system into the nectar and pollen which is one main exposure route for pollinators (Bogdanov 2006; Silici et al. 2013; Hladun et al. 2015). Cd is more mobile in plants than most other metals (Verkleij and Schat 1990), and a correlation of Cd concentrations in *Trifolium pratense* flowers and honey was found (Leita et al. 1996). Thus, this exposure path might have also been one of the main sources of Cd exposure to bumblebees in our study.

Cu concentrations vary between 10 mg/kg (Goretti et al. 2020) and 38 mg/kg dw (Veleminski et al. 1990) in studies which used honeybees as bioindicators. These values are lower compared to the maximum concentration of 47 mg/kg we found at one urban site. A lot of different land-use types have been analysed using honeybees and even highly industrialised areas show comparatively low Cu concentrations (see e.g. Veleminski et al. 1990) compared to concentrations found at our sampling sites which are far away (~100 km) from industry or coal combustion emitters.

Literature Ni concentrations in honeybees vary between 0.19 mg/kg at a rural site in the Netherlands (Van der Steen et al. 2012) and 7.5 mg/kg in an highly industrialised area in Italy (Goretti et al. 2020). Maximum Ni values of 2.6 mg/kg in this study were found at an agrarian site, which is high compared to most other studies which found maximum values of 1.48 mg/kg at various different land-use types such as urban, industrialised and agricultural-forest regions (Roman 2005; Van der Steen et al. 2012, 2016; Zarić et al. 2017).

Hg was the metal found at lowest concentrations of 0.002 to 0.02 mg/kg in bumblebees in this study. This is within the concentration range of 0.008 to 0.04 mg/kg, found in a study from Slovakia using honeybees (Toth et al. 2016). Another study from Slovakia from Toporcák et al. (1992) shows high concentrations of up to 3.42 mg/kg found in honeybees collected in industrially contaminated areas. They analysed different parts of the body separately and found the following concentrations: in heads, 0.029–0.385 mg/kg; thorax, 0.028–0.595 mg/kg; and abdomen, 0.083–2.255 mg/kg. For Hg, the obtained results should only be seen as a baseline to estimate the minimum Hg concentration, as possible losses during the drying process are not considered. However, according to Hojdovà et al. (2015) (who measured Hg losses in soils), Hg losses during the drying process should not exceed 8%.

As a first conclusion, metal concentrations found in bumblebees mostly range within the same order of magnitude as those in honeybees. Thus, the bumblebee *Bombus terrestris* is at least as suitable as the honeybee as a metal bioindicator. One advantage of the bumblebee is that it can be used in colder regions where honeybees naturally do not exist and can thus provide information on metal pollution in special areas like the Himalaya or even the arctic region (Goulson 2006).

It was surprising that some metals (such as Cu, Ni, As or Cr) were found in relatively high concentrations compared to concentrations found in honeybees sampled in highly polluted areas (e.g. heavy industry). According to information from the lower Immission Control Authority in Aachen, no companies are located in the city which emit heavy metals in high amounts. Bigger emitters such as metal processing companies are located in Stolberg, around 10 km away from Aachen so that due to atmospheric deposition, metals emitted there could reach Aachen. In 2020, elevated metal concentrations were measured within the air reaching concentrations above threshold values for the prevention of human health (Mirgel and Wruck 2020).

It is difficult to make a statement about the threat of metals for bumblebees in the concentration range found in this study as data on other species than honeybee is still scarce. Furthermore, mixture toxicity of various metals for insects is largely unresearched (Burden et al. 2019). Bumblebees, as one of the most important pollinator groups, should be further considered in toxicity tests but also as additional bioindicators besides honeybees due to their lifecycle and behaviour.

### Inter-sample variation

Metal concentrations in part vary a lot among the samples. Even samples from the same location and the same sampling day vary in their concentrations of up to one order of magnitude leading to high standard deviations (SI, table S2). This is in accordance with results from Zarić et al. (2016) and Zarić et al. (2022b) who found high standard deviations for metals within honeybees and explained this with bees flying in different directions to sites with different levels of contamination.

Bumblebees were sampled when returning to the hives so that we are sure that the captured individuals left the hive at least once and got in contact with the “outdoor” environment. But several uncertainties remain which might influence variation in metal concentrations even within replicates. One important factor, which is difficult to assess, is the age of every individual as this influences the number of forage flights which have been conducted before it was captured. It was shown by Leita et al. (1996) that the time of exposure correlates with the metal accumulation in honeybee bodies. This is likely to be the case for bumblebees as well. Furthermore, it is unknown in which direction and which distances every individual has flown before the catch. Therefore, a great variance in foraging time and environmental exposure is assumed.

Significantly higher concentrations for both land use types (urban and agrarian) compared to the control samples were found for Fe, Cu, B, Al and Pb. Most of the remaining metals also seem to have higher concentrations at both land use types, but the difference was not significant. One reason for that could be the low number of replicates as well as the high variability in metal concentrations within the same hives which leads to a high standard deviation.

Another reason might be the ability of bumblebees to regulate the internal metal concentration for some metals such as the essential Zn. As a trace metal, it is essential for the survival of most organisms as it maintains biological functions. In the given study, we did not find significant differences in Zn concentration between the control and field samples. These results are in accordance with Szentgyorgyi et al. (2011) who investigated Zn concentrations along a pollution gradient (measured as distance from a Zn smelter) in different *Bombidae* species. They found varying Zn concentrations in soil samples among the gradient but constant Zn levels within the different *Bombidae* species. This could be due to its presence in metallothioneins, proteins responsible for the regulation of metal homeostasis and detoxification (Kägi and Schäffer 1988; Purać et al. 2019). These proteins control metal concentrations within the body either by deposition of metals within parts of the body as inactive, non-toxic species or by active excretion (e.g. in faeces) (Szentgyorgyi et al. 2011). The existence of metallothioneins is not yet confirmed for bumblebees but known for honeybees (Purać et al. 2019). However, our findings suggest that *B*. *terrestris* has some regulatory mechanisms to maintain constant Zn concentrations within their bodies even in changing environmental concentrations.

### Spatial differences—agrarian and urban sites

Comparing mean values of all agrarian sites with those of all urban sites (including samples from April and July), significant differences in concentrations were only found for two out of the twelve metals analysed: B and Cd showed significantly higher concentrations in samples from agrarian sites. B, an essential micronutrient for plants, is one component used in fertilisers especially in foliar fertilisers for rape fields (Siede et al. 2013). One of our agrarian sampling sites was located next to a rape field which could have been treated with boron fertiliser.

Cd is considered as one of the major threats in soils which can pose a serious health risk when adsorbed into food crops. High Cd levels in soils originate either from the parent material or from anthropogenic input, in agricultural areas mainly due to sewage sludge and phosphate fertiliser application (Kabata-Pendias 2011). Soil erosion might be higher at agrarian sites than at urban sites as most of the surfaces in urban regions are sealed, whereas agrarian sites are characterised by huge areas of bare soil, especially before sowing when no vegetational cover exists. Zuazo et al. (2011) could show that plant covers prevent soil erosion and therefore decrease heavy metal release into the environment compared to bare soils.

Main anthropogenic metal sources into agrarian soils are as follows: animal manure, mineral fertilisers and pesticides as well as other agrochemicals, irrigation water and atmospheric deposition (Belon et al. 2012; Hou et al. 2014; Shi et al. 2018; Hou et al. 2014). One possible explanation for the higher concentrations at the agrarian sites (in April) could be the application of metal containing agrochemicals or phosphate fertilisers or sewage sludge before sowing. There is a high variation within metal concentrations in sewage sludge, depending on the type of sewage treatment plant and the origin of the wastewater (e.g. industrial or domestic, (Alloway 2013)).

Especially, the site located on the ground of the apple orchard shows high concentrations of Cd, Ni, Cr, Zn and Fe compared to the other agrarian sites. Apple orchards are upon the cultivars with highest application rates of agrochemicals with an average number of 21 applications per season in Germany (Sybertz et al. 2020). Especially in spring time, high amounts of different pesticides are applied to the orchards (Sybertz et al. 2020).

Van der Steen et al. (2016) investigated honeybees from 150 different land-use types in the Netherlands. Their results agree with our findings as higher metal concentrations were found in agrarian than in urban sites. As they did not have an explanation for this finding, they recommended further studies on land use effects to reveal the mechanisms resulting in different metal concentrations found in bees. One hypothesis could be that bumblebees are more active outside the cities and conduct more forage flights so that they would collect more pollutants.

### Temporal differences April and July

The mean total metal concentration of all samples, taken in April, was 259 mg/kg dw, whereas the one taken in July was 384 mg/kg dw, indicating that exposure of bumblebees was higher in July than in April. This difference was highly significant (*p* < 0.005) and relates to Fe and Zn concentrations. These two metals constitute between 78 and 85% of the total metal concentration at the agrarian sites and in the control, respectively. The source for this increased metal load is difficult to identify as no large Fe or Zn emitter is located in or close to Aachen. Significant differences in concentration by comparing all April and July samples were also found for B, Cu, Ni, Cd, As and V. Van der Steen et al. (2012) also investigated temporal differences in honeybees (2-week sampling) and found significant differences for Al, Cd, Cr, Cu and V but not for As, Ni, Pb and Zn.

 Considering the results of both land-use types separately, especially the strong threefold increase in the cadmium concentration at the urban sites from April to July, is striking. Our results indicate the seasonal variation in exposure of bumblebees to these metals. Reasons for such fluctuations might be differences in metal emissions for example in traffic or in the above-mentioned soil erosion which in early spring is higher than in summer since the vegetational cover is not yet fully grown.

Another possibility is the change in bumblebee activity. In July, where most of the flowers have their flowering peak (Baude et al. 2016), it is also peak time for collecting nectar and pollen. Bumblebees have their highest nectar demand in August (Jachuła et al. 2021) leading to the assumption that their foraging activity, and thus, exposure is also highest in these months.

Another explanation for the higher metal concentrations found in July could be the decreased precipitation in summer causing maximum re-suspension of air-borne dust particles from various sources (Alloway 2013). Soil moisture contributes to bind particles together; thus, wind erosion decreases with higher soil moisture (Laidlaw and Filippelli 2008). Various studies have shown that the concentration of soil moisture is an important control of dust (PM10) suspension and loading (Nickovic et al. 2001; Cornelis and Gabriels 2003; Harris and Davidson 2005; Laidlaw and Filippelli 2008). Metals can attach to dust particles such as the PM_10_ fraction and accumulate for example within road dusts (Duong and Lee 2011) which could also be the source of the high metal concentrations found in July.

Vanadium showed a significantly higher concentration at the agrarian sites in April than in July, these results agree with findings from Anke (2004) who described decreasing V concentrations from the beginning of May to the middle of June of up to a third of the initial level in plants. Wang et al. (2020) found highest V concentrations in aerosols (PM_10_ fraction) around a V smelter in spring and lowest in summer. They suggested that this could be due to seasonal periodicity of atmospheric conditions.

### The Honeybee Contamination Index

Based on the established HCI, we here develop the corresponding index for bumblebee contamination. It was surprising that at the sampling site A1 (orchard), a high level of pollution was found, whereas the urban site U3, located at the Blücherplatz within the city of Aachen, showed a low mean level of pollution in April. It was expected that the urban sites would show a higher pollution level than the agrarian sites as more possible metal sources such as fuel combustion or tyre and brake wear are found in urban sites. The possible reasons for the increase of metal pollution from spring to summer were discussed before in detail.

## Conclusion

The results found in this study show that the native bumblebee *Bombus terrestris* is a suitable bioindicator to detect temporal and spatial patterns in environmental metal concentrations. All twelve target metals could be found above the detection limits, and the concentrations are within the range found in other studies which used honeybees as bioindicators. Compared to honeybees, bumblebees have several advantages for monitoring pollutants like their adaptation to colder temperatures, allowing foraging flights earlier and later during the day, and smaller flight radii which leads to a higher spatial resolution for monitoring.

Significant differences in metal concentrations between urban and agrarian sites were detected for Cd and B. High inter-sample variability was observed, likely resulting from spatial and temporal differences in the activity of individual bumblebees. Further studies should consider pooling higher numbers of bumblebees from the same hives together to cover a wider range of different movement patterns and ages. It would be interesting to sample honeybees and bumblebees from the same locations to compare metal burden to evaluate the representativeness of these species for other insect species.

### Supplementary Information

Below is the link to the electronic supplementary material.

Supplementary file1 (DOCX 421 KB)

## Data Availability

Data is available on demand from the authors.
