# Supplementary material for: Monitoring metal patterns from urban and agrarian sites using the bumblebee Bombus terrestris as a bioindicator
Source: Environ Sci Pollut Res Int. 2023 Nov 7;30(57):119947–60. doi: 10.1007/s11356-023-30504-w (PMC10698117; doi:10.1007/s11356-023-30504-w)
Supplement: Supplementary file 1 — Supplementary file1 (DOCX 421 KB) [file 11356_2023_30504_MOESM1_ESM.docx]

Supplementary Material

***Table S1****: Overview of samples taken per site and sampling date and per control.*

| **Site** | **April** | **July** |
| --- | --- | --- |
| A1 | 2 | 2 |
| A2 | 3 | 2 |
| A3 | 3 | 2 |
| U1 | 2 | 2 |
| U2 | 2 | 2 |
| U3 | 3 | 2 |
| **total** | **15** | **12** |
| Control NL | 2 | |
| Control GE | 1 | |

***Table S2****: Trace metal concentrations in mg/kg dry weight bumblebee found in the control (contr) or at either the agrarian (A) or urban (U) site, at sampling date in April or July (indicated with the Roman II behind the site name). Shown are the mean values and standard deviations (SD) for Iron (Fe), Zinc (Zn), Copper (Cu) Boron (B) Chromium (Cr) and Vanadium (V). The highest concentration found for the respective metal is marked in red, the lowest is marked in green.*

| **Site** | **Fe** | | **Zn** | | **Cu** | | **B** | | **Cr** | | **V** | |
| --- | --- | --- | --- | --- | --- | --- | --- | --- | --- | --- | --- | --- |
|  | **mean** | **SD** | **mean** | **SD** | **mean** | **SD** | **mean** | **SD** | **mean** | **SD** | **mean** | **SD** |
| **Contr** | 74.18 | 27.04 | 77.67 | 46.73 | 11.47 | 4.41 | 8.13 | 2.37 | 0.42 | 0.07 | 0.07 | 0.06 |
| **A1** | 112.25 | 0.57 | 108.80 | 33.16 | 13.42 | 0.85 | 22.41 | 2.01 | 1.28 | 0.34 | 0.20 | 0.00 |
| **A1 II** | 169.94 | 16.02 | 151.95 | 9.26 | 29.70 | 0.25 | 13.86 | 1.83 | 1.44 | 0.31 | 0.02 | 0.00 |
| **A2** | 104.19 | 12.72 | 96.04 | 17.33 | 24.75 | 4.12 | 28.83 | 6.64 | 0.82 | 0.21 | 0.03 | 0.00 |
| **A2 II** | 157.95 | 27.62 | 125.86 | 16.79 | 23.79 | 5.32 | 18.67 | 1.31 | 0.41 | 0.04 | 0.03 | 0.00 |
| **A3** | 134.93 | 29.99 | 106.49 | 3.53 | 23.36 | 2.91 | 33.88 | 3.54 | 0.73 | 0.10 | 0.13 | 0.13 |
| **A3 II** | 159.91 | 5.47 | 115.45 | 8.33 | 23.72 | 1.85 | 12.18 | 1.97 | 0.42 | 0.00 | 0.03 | 0.00 |
| **U1** | 94.43 | 32.48 | 77.25 | 17.94 | 19.57 | 6.31 | 14.95 | 1.38 | 0.87 | 0.25 | 0.17 | 0.03 |
| **U1 II** | 219.62 | 11.96 | 162.66 | 6.06 | 39.63 | 10.30 | 15.01 | 4.54 | 0.78 | 0.19 | 0.04 | 0.00 |
| **U2** | 95.05 | 5.68 | 95.84 | 13.08 | 26.58 | 1.32 | 17.69 | 1.30 | 0.61 | 0.01 | 0.02 | 0.00 |
| **U2 II** | 173.77 | 32.84 | 134.39 | 14.86 | 25.74 | 5.74 | 14.24 | 0.79 | 0.47 | 0.12 | 0.03 | 0.00 |
| **U3** | 78.64 | 30.93 | 70.91 | 23.43 | 20.39 | 6.31 | 16.79 | 5.48 | 0.47 | 0.20 | 0.18 | 0.06 |
| **U3 II** | 219.92 | 43.83 | 137.91 | 5.73 | 30.01 | 4.15 | 15.50 | 1.65 | 0.64 | 0.03 | 0.07 | 0.04 |

***Table S3****: Heavy metal concentrations in mg/kg dry weight bumblebee found at either the agrarian (A) or urban (U) sites, at sampling date April or July (indicated with the Roman II behind the site name). Shown are the mean values and standard deviations (SD) for Aluminium (Al), Nickel (Ni), lead (Pb), Cadmium (Cd), Arsenic (As) and mercury (Hg). The highest concentration found for the respective metal is marked in red the lowest is marked in green.*

| **Site** | **Al** | | **Ni** | | **Pb** | | **Cd** | | **As** | | **Hg** | |
| --- | --- | --- | --- | --- | --- | --- | --- | --- | --- | --- | --- | --- |
|  | **mean** | **SD** | **mean** | **SD** | **mean** | **SD** | **mean** | **SD** | **mean** | **SD** | **mean** | **SD** |
| **Contr** | 4.93 | 1.25 | 0.39 | 0.07 | 0.15 | 0.1 | 0.1 | 0.1 | 0.13 | 0.14 | 0.01 | 0.01 |
| **A1** | 21.84 | 3.51 | 1.72 | 1.30 | 0.76 | 0.32 | 0.54 | 0.07 | 0.11 | 0.00 | 0.02 | 0.00 |
| **A1 II** | 10.66 | 0.12 | 1.00 | 0.18 | 0.60 | 0.48 | 0.94 | 0.01 | 0.15 | 0.01 | 0.01 | 0.00 |
| **A2** | 9.72 | 1.08 | 0.50 | 0.06 | 0.72 | 0.29 | 0.27 | 0.08 | 0.12 | 0.01 | 0.01 | 0.00 |
| **A2 II** | 13.16 | 0.87 | 0.54 | 0.12 | 0.44 | 0.20 | 0.51 | 0.06 | 0.18 | 0.03 | 0.02 | 0.00 |
| **A3** | 31.61 | 22.13 | 0.47 | 0.08 | 1.74 | 1.58 | 0.27 | 0.08 | 0.16 | 0.03 | 0.02 | 0.00 |
| **A3 II** | 11.47 | 2.14 | 0.55 | 0.06 | 0.29 | 0.06 | 0.35 | 0.00 | 0.10 | 0.00 | 0.01 | 0.00 |
| **U1** | 9.06 | 4.18 | 0.57 | 0.14 | 0.35 | 0.09 | 0.07 | 0.02 | 0.14 | 0.07 | 0.02 | 0.01 |
| **U1 II** | 21.00 | 2.55 | 0.83 | 0.23 | 0.69 | 0.35 | 0.38 | 0.16 | 0.23 | 0.03 | 0.02 | 0.01 |
| **U2** | 6.82 | 2.76 | 0.36 | 0.07 | 0.54 | 0.25 | 0.10 | 0.04 | 0.14 | 0.02 | 0.01 | 0.00 |
| **U2 II** | 13.49 | 1.12 | 0.53 | 0.04 | 0.59 | 0.09 | 0.25 | 0.00 | 0.15 | 0.01 | 0.02 | 0.00 |
| **U3** | 8.49 | 3.67 | 0.55 | 0.34 | 0.34 | 0.17 | 0.07 | 0.02 | 0.11 | 0.04 | 0.01 | 0.01 |
| **U3 II** | 24.60 | 9.68 | 0.68 | 0.13 | 0.65 | 0.10 | 0.32 | 0.06 | 0.22 | 0.02 | 0.02 | 0.00 |

***Figure S1****: Mean lead concentrations [mg/kg dw] and standard deviations, found in vegetation, soil and bumblebee samples on the three agrarian sites (A1, A2 and A3).*

***Table S4:*** *Main Emission sources as percent on total emission at the different sites. Data adapted and modified from: Jens Feigenspan (UBA 2020)*

| **site** | **As** | | **Cd** | | **Pb** | | **Ni** | | **Hg** | |
| --- | --- | --- | --- | --- | --- | --- | --- | --- | --- | --- |
|  | **source** | **%** | **source** | **%** | **source** | **%** | **source** | **%** | **source** | **%** |
| **A2** | agriculture | 72 | agriculture | 66 | agriculture | 96 | agriculture: off-road vehicles | 69 | agriculture: off-road vehicles | 70 |
| **A2** | Military vehicles | 18 | agriculture: off-road vehicles | 34 | Military vehicles | 3 | Military vehicles | 17 | agriculture | 25 |
| **A3** | Automobile tyre and brake wear | 92 | Automobile tyre and brake wear | 90 | Automobile tyre and brake wear | 99 | Automobile tyre and brake wear | 80 | Fuel combustion in passenger cars | 72 |
| **A3** | Automobile road abrasion | 7 | agriculture | 5 |  |  | Automobile road abrasion | 17 | Road transport | 12 |
| **U1** | Copper production | 33 | Copper production | 37 | Other product use | 36 | Railways | 83 | Stationary combustion | 30 |
| **U1** | Public electricity and heat production | 22 | Other product use | 31 | Automobile tyre and brake wear | 23 | Other product use | 8 | Public electricity and heat production | 21 |
| **U2** | Copper production | 29 | Other product use | 38 | Other product use | 41 | Other product use | 46 | Stationary combustion | 37 |
| **U2** | Stationary combustion | 20 | Copper production | 31 | Automobile tyre and brake wear | 19 | Iron and steel production | 15 | Public electricity and heat production | 18 |
| **U3** | Copper production | 50 | Copper production | 62 | Iron and steel production | 45 | Iron and steel production | 41 | Public electricity and heat production | 37 |
| **U3** | Public electricity and heat production | 33 | Iron and steel production | 12 | Other product use | 13 | Public electricity and heat production | 31 | Iron and steel production | 24 |
